# Electroconvulsive Therapy in a Patient with Somatic Symptom Disorder and Major Depression: A Case Report

**DOI:** 10.1192/j.eurpsy.2026.11236

**Published:** 2026-07-31

**Authors:** H. G. Kurt, B. Koparal

**Affiliations:** Department of Psychiatry, Gazi University Faculty of Medicine, Ankara, Türkiye

## Abstract

**Introduction:**

According to the Diagnostic and Statistical Manual of Mental Disorders, Fifth Edition (DSM-5), **Somatic Symptom Disorder (SSD)** is defined by one or more somatic symptoms associated with excessive thoughts, feelings, or behaviors related to the symptoms or associated health concerns. Pain, defined as an unpleasant sensory and emotional experience associated with actual or potential tissue damage, commonly co-occur with depressive symptoms. The comorbidity between SSD and Major Depressive Disorder (MDD) has been reported in approximately 50–70% of cases.

**Objectives:**

This case emphasizes the importance of recognizing the shared affective and neurobiological mechanisms underlying pain and depression, and the potential therapeutic role of Electroconvulsive Therapy (ECT) in selected SSD patients with treatment-resistant depressive features.

**Methods:**

Description of a clinical case and literature review of the current literature.

**Results:**

A 32-year-old woman was hospitalized with a 9-month history of **bruxism, and severe orofacial pain,** excessive health-related preoccupations, pain related depressive symptoms and suicidal thoughts.

Her psychiatric history included burning abdominal pain and somatic delusions following sexual trauma at age 26, which partially remitted with sertraline (100 mg/day) and aripiprazole (5 mg/day).

Prior to admission, dental and neurological evaluations, including MRI imaging, did not reveal sufficient pathology to explain the symptoms.

During the hospitalization, she was treated with **sertraline 150 mg/day, aripiprazole 5 mg/day first. Then she received 20 sessions of repetitive Transcranial Magnetic Stimulation (rTMS)**, with no improvement. Due to a lack of response to current medications, treatment changed to **venlafaxine (225 mg/day)** and **olanzapine (10 mg/day)**. After that, she received **12 sessions of bilateral ECT because of severe and persistent suicidal thoughts and attempts.**

**After ECT, pain intensity and depressive symptoms noticeably decreased, both pain tolerance and daily functioning improved.** No cognitive or neurological adverse effects were observed.

**Conclusions:**

ECT may improve both affective and somatic symptoms in SSD with comorbid depression by modulating shared monoaminergic and limbic–cortical pathways. This case supports ECT as a valuable treatment option for **refractory SSD with MDD comorbidity**, especially when conventional pharmacotherapy and rTMS are ineffective.

**To our knowledge, this is one of the few reported cases demonstrating improvement of chronic orofacial pain and depressive symptoms following ECT in a patient with SSD.**

**Disclosure of Interest:**

None Declared

